# Distinct trajectories of perinatal depression in Chinese women: application of latent growth mixture modelling

**DOI:** 10.1186/s12884-021-04316-0

**Published:** 2022-01-10

**Authors:** Lan Hong, Tao Le, Yinping Lu, Xiang Shi, Ludan Xiang, Meng Liu, Wenmiao Zhang, Meixi Zhou, Jiangling Wang, Dongwu Xu, Xin Yu, Ke Zhao

**Affiliations:** 1 School of Mental Health, Wenzhou Medical University, Wenzhou, 325035 China; 2 Department of Obstetrics, First Affiliated Hospital of Wenzhou Medical University, Wenzhou, China; 3 The Affiliated Kangning Hospital of Wenzhou Medical University, Wenzhou, 325035 China

**Keywords:** Longitudinal trajectories, LGMM, Perinatal depression, Social support

## Abstract

**Background:**

Current research on perinatal depression rarely pays attention to the continuity and volatility of depression symptoms over time, which is very important for the early prediction and prognostic evaluation of perinatal depression. This study investigated the trajectories of perinatal depression symptoms and aimed to explore the factors related to these trajectories.

**Methods:**

The study recruited 550 women during late pregnancy (32 ± 4 weeks of gestation) and followed them up 1 and 6 weeks postpartum. Depressive symptoms were measured using the Edinburgh Postnatal Depression Scale (EPDS). Latent growth mixture modelling (LGMM) was used to identify trajectories of depressive symptoms during pregnancy.

**Results:**

Two trajectories of perinatal depressive symptoms were identified: “decreasing” (*n* = 524, 95.3%) and “increasing” (*n* = 26, 4.7%). History of smoking, alcohol use and gestational hypertension increased the chance of belonging to the increasing trajectories, and a high level of social support was a protective factor for maintaining a decreasing trajectory.

**Conclusions:**

This study identified two trajectories of perinatal depression and the factors associated with each trajectory. Paying attention to these factors and providing necessary psychological support services during pregnancy would effectively reduce the incidence of perinatal depression and improve patient prognosis.

**Supplementary Information:**

The online version contains supplementary material available at 10.1186/s12884-021-04316-0.

## Introduction

The World Health Organization (WHO) widely defines perinatal depression (PND) as a severe depressive episode during pregnancy or a year after childbirth. The high prevalence of PND is a global phenomenon [1]. In high-income countries, the prevalence of antenatal and postnatal depression varies between 7 and 15% [2], and between 19 and 25% in low-income countries [3]. PND is considered one of the most common complications of pregnancy and has a significant impact on the mother, her offspring and even her entire family [4, 5]. When not treated properly, PND has a negative impact on the outcome of childbirth [6], the child’s health, and their social and emotional development [7]. Previous theoretical developments have revealed that children whose mothers suffered from PND have a significantly higher risk of depression compared to children whose mothers did not experience PND, and this risk persists from childhood through to adolescence and adulthood. For children of mothers with PND, the probability of being diagnosed with depression in adolescence and adulthood, respectively, is almost 1.28 times and 3.4 times greater than that of children whose mothers did not have PND [8, 9]. Even more seriously, the adverse effects of PND are far-reaching and intricate, lasting for multiple generations; this is referred to as intergenerational transmission [10, 11].

Previously, most studies of PND were cross-sectional in nature. The typical strategy used to evaluate the severity of depressive symptoms was to categorise thresholds of severity at a single time point [12]. Some scholars argue that the symptoms of depression are diverse and can differ with respect to onset, course, duration and severity [13–15]. Thus, it is difficult to fully explore the causal factors involved in the onset and variation in depressive symptoms when considering a depressive episode as a point rather than a continuous line and ignoring the continuity and fluctuations in the disorder [16, 17]. Researchers widely consider that PND can occur from the first trimester to one year after delivery [18]. There are significant differences in the incidence of depression in the different periods of pregnancy. The prevalence of depressive symptoms in the first and second trimesters is much lower than in the third trimester [19]. Due to the long duration of pregnancy, women can experience mood fluctuations throughout the various stages of pregnancy [20]. Some studies have shown that diverse individual characteristics play a significant role in the onset, course, duration and severity of the disease [13–15]. Early surveillance of these fluctuations, and early behavioural intervention and psychological counselling will have a positive impact on the outcomes of perinatal depression. This could also help clinicians to identify at risk groups of women, who may experience adverse outcomes in the future (even in the early stages of pregnancy) [21, 22]. It is essential to understand the occurrence, development and variation in PND throughout the perinatal period and implement interventions as early as possible. Longitudinal mixed effect and growth mixed models are usually used to evaluate the development of perinatal depression symptoms [23]. Variation in the progression or trajectory of symptoms is assessed, and potential subgroups are identified; these can explain the different characteristics of depression symptoms in the different potential groups [24].

Several different PND trajectories have been reported [22, 25], but there is considerable heterogeneity in the reports. A meta-analysis of 23 studies related to PND symptom trajectories found that some research teams determined the trajectory of depression based on the characteristics and severity of the disease [26]. In contrast, others chose advanced statistical methods [27–30]. Thus, the variation in the number of final trajectories reported in different studies, ranging from two to six different PND trajectories, is likely related to the different grouping standards. Further, the various reported trajectories are inconsistent and sometimes even contradictory. Many predictors attributed to a high symptom level trajectory have been identified, for example, younger age, low education level, unemployment and low-level social support [26, 31]. Other studies have reported that pregnancy complications increase the possibility of a persistent depression trajectory. The persistent depression trajectory is also associated with the maternal preterm delivery than other trajectories [27]. There is less published evidence for a dynamic trajectory of depression in the Asian population.

The multiple trajectories in the development of depressive symptoms indicate that there may be specific risk factors that lead to particular patterns of symptoms. Exploration of depression trajectories and their predictive factors will assist in the detection of positive cases [32], facilitate timely targeted interventions, and offer individualized treatments. To this end, the current study aimed to provide new evidence on PND dynamic trajectories in the Asian population while also exploring changes in the trajectory of depressive symptoms and evaluating related predictive factors. In particular, the focus was on the influence of factors such as social support on PND trajectories. The following hypotheses were formulated:

- Depressive symptoms of pregnant women in the perinatal period have different development trajectories.
- Perinatal depression is related to a variety of social and environmental factors.

Validation of these hypotheses will contribute to an improved understanding of perinatal depression and will provide guidance for psychological intervention and support.

## Method

This study was part of a longitudinal research project on the prenatal and postnatal mental health of pregnant and lying-in women at the First Affiliated Hospital of Wenzhou Medical University. The study was conducted from January 2018 to January 2019.

### Setting and participants

**Fig. 1:**
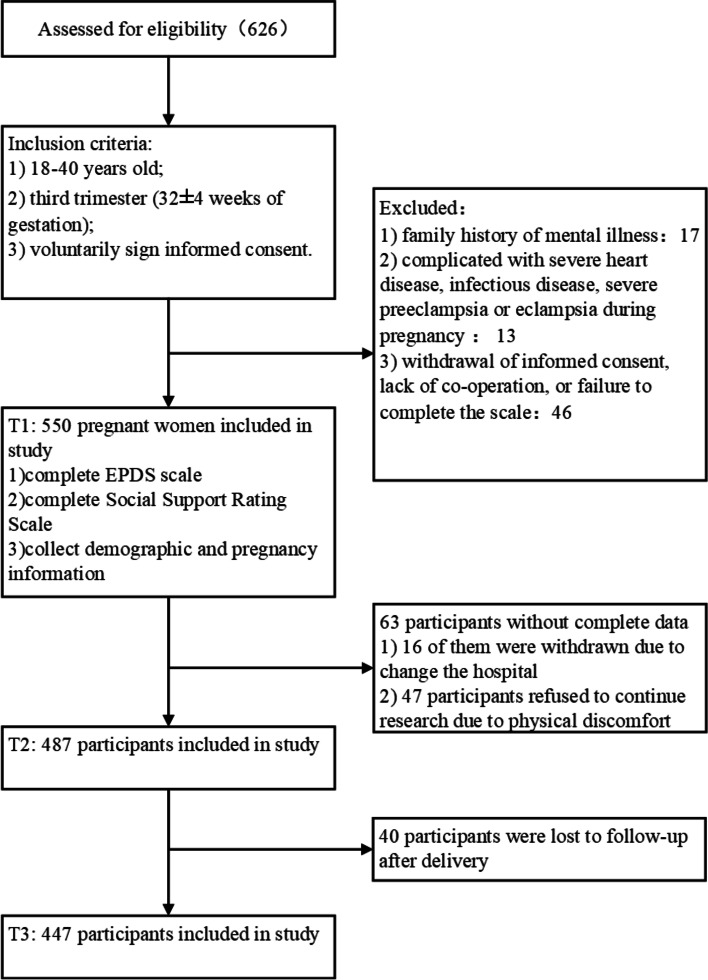
Sample flow chart. T1: Third trimester (32 ± 4 weeks of gestation), T2: 1 week postpartum, T3: 6 weeks postpartum

The Department of Obstetrics of the First Affiliated Hospital of Wenzhou Medical University participated in the current study. The annual obstetric outpatient volume of this hospital exceeds 110,000. In total, 626 women were randomly selected from the outpatient clinic, of whom 550 met the inclusion criteria for this study. Of these, 63 women were lost in the first follow-up and 40 were lost in the second follow-up. Analyses in this paper were conducted in 550 women. Detailed information regarding participation at each follow-up is presented in Fig. 1.

A group of professionally trained investigators assisted the participants to complete the self-report survey. The researchers explained the research content to the participants before the participants voluntarily provided written informed consent. The questionnaires were completed anonymously and were numerically coded. Participants were also informed that they could withdraw at any time. After each participant completed the survey, the researchers immediately reviewed their results.

### Procedure

The Edinburgh Postnatal Depression Scales (EPDS) was administered at three time points. The first time point was during the prenatal period, within the third trimester (gestational week 32 ± 4, T1). The other two time points were during the postnatal period, at 1 week (T2) and 6 weeks postpartum (T3). In addition to the EPDS, participants completed demographic questions and the Social Support Rating Scale (SSRS) at T1. The first follow-up (T2) was conducted on the ward. The second follow-up (T3) was conducted during the participant’s outpatient review. Three days before their scheduled outpatient obstetric appointment, the researcher called the participant to confirm the date of their review and to remind them that they would be completing their second follow-up survey at this review appointment.

The inclusion criteria for the study were as follows: 1) 18–40 years old; 2) third trimester (32 ± 4 weeks of gestation); and 3) voluntarily signed informed consent.

The exclusion criteria were: 1) family history of mental illness; 2) severe heart disease, infectious disease, severe preeclampsia, or eclampsia during pregnancy; and 3) withdrawal of informed consent, lack of co-operation or failure to complete the scale.

### Measures

#### Self-report demographic survey

Under the supervision of trained evaluators, the participants filled out the social and demographic section of the survey: age, location (city, rural), years of education (≤9 years, 10–12 years, 13–16 years), monthly income (low: < ¥5000), high: ≥ ¥5000), abortion history (Yes, No), behavioural factors (history of smoking, history of alcohol and exercise during pregnancy - (Yes, No)), pregnancy complications (gestational diabetes and pregnancy-induced hypertension - (Yes, No)).

#### EPDS

The Chinese equivalent of the EPDS [33] was used to quantitatively assess participants’ depression symptoms at enrolment (T1), 1 week after delivery (T2), and 6 weeks after delivery (T3). The scale was developed by Cox et al. [34] and assesses three dimensions, emotional loss, anxiety, and depression. The scale comprises 10 items, each item is scored according to the severity of symptoms (0–3 points), with total possible scores ranging from 0 to 30 points. The higher the score, the more severe the symptoms of depression [35]. This scale has good reliability and validity in mainland China [36]. The EPDS has complete measurement equivalence between prenatal and postpartum females; it can be used as a prenatal screening test and is a useful tool for depression treatment [37].

#### SSRS

The Chinese equivalent of the SSRS, developed by Xiao Shuiyuan in 1994 [38], was used to assess participants’ social support status. The SSRS measures three components, objective support, subjective support, and support utilization, and comprises 10 items. Each item is assessed on a Likert scale. The scale has good reliability and validity [39]. The higher the score, the more social support available to the participant.

### Statistical analyses

First, missing values for the covariates were imputed. For age and years of education there were 9 (1.6%) and 12 (2.2%) women with missing values, respectively. They were imputed with the sample. The missing values for SSRS (33 women) were replaced by the mean. Descriptive analysis (means, standard deviations, counts and percentages) was performed to examine the distributions of the variables.

Second, we explored different development trajectories of perinatal depression via latent variable mixed growth model (LGMM). The missing values were processed by full information maximum likelihood procedure (FIML). Given the skewed count-type nature of the EPDS data (see Additional file 1), we specified a Poisson distribution for the outcome variable. To represent non-equidistant time points between the assessments, factor loadings were fixed to 0, 1 and 1.5 to represent assessments at baseline, and then one- and six-weeks postpartum. Random intercept variances were allow to vary across classes. This model was estimated from 1 up to 4 latent classes, using 200 random starting values. Selection of number of classes was decided based on the Akaike information criterion (AIC) [40] and Bayesian information criterion (BIC) [41]. The smaller the AIC and BIC values, the better the fit of the model. The bootstrapped likelihood ratio test (BLRT) and Vuong-Lo-Mendell-Rubin test (VLMR) were also considered. A significant likelihood ratio test for k classes with *p* < 0.05 indicates that the specified k-class model is an improvement over a model with k-1 classes [42]. The entropy value indicates the accuracy with which the model can classify individuals into their corresponding classes; it ranges from 0 to 1. Entropy values ≥0.8 indicate that the classification accuracy exceeds 90% [43]. In cases where fit indices between the two models were relatively similar, entropy values were also taken into account. Finally, the size and theoretical interpretability of the classes were also considered. The LGMM model was estimated with Mplus version 8.0 [44]. The Mplus syntax can be found in Additional file 2.

Third, participants were assigned to latent classes based on their highest posterior probability. Simple logistic regression (using only one predictor variable at a time) was used to explore the association between social and environmental factors with the trajectory class membership. The logistic regression model was performed using SPSS version 24.0. Odds ratios (OR) with 95% confidence intervals are reported, with a significance level set at 5%.

## Results

### Overview of the sample

Descriptive statistics for the sample are presented in Table 1. On average, participants were 28.7 years of age (SD = 4.1). The majority of participants reported finishing college school (*n* = 359, 65.3%), living in rural (*n* = 358, 65.1%), and monthly income ≥¥5000(*n* = 433, 78.7%). Additionally, the mean values of EPDS at the three time points were 7.6 (SD = 3.7), 6.4 (SD = 3.5), 6.2 (SD = 4.5).

**Table 1:** Characteristics of the sample, by class

Demographics	Total (*n* = 550)		Decreasing (*n* = 524, 95.3%)		Increasing (*n* = 26, 4.7%)	
	(n, %)	Mean (SD)	n (%)	Mean (SD)	n (%)	Mean (SD)
Age		28.7 (4.1)		28.7 (4.1)		29.4 (4.1)
Location						
City	192 (34.9)		183 (34.9)		9 (34.6)	
Rural	358 (65.1)		341 (65.1)		17 (65.4)	
Years of education						
≤ 9 years	72 (13.1)		68 (13.0)		4 (15.4)	
10–12 years	119 (21.6)		116 (22.1)		3 (11.5)	
13–16 years	359 (65.3)		340 (64.9)		19 (73.1)	
Monthly income						
<¥ 5000	117 (21.3)		109 (20.8)		8 (30.8)	
≥ ¥5000	433 (78.7)		415 (79.2)		18 (69.2)	
History of smoking						
No	537 (97.6)		514 (98.1)		23 (88.5)	
Yes	13 (2.4)		10 (1.9)		3 (11.5)	
History of alcohol						
No	519 (95.3)		497 (94.8)		22 (84.6)	
Yes	31 (4.7)		27 (5.2)		4 (15.4)	
Exercise during pregnancy						
No	478 (86.9)		455 (86.8)		23 (88.5)	
Yes	72 (13.1)		69 (13.2)		3 (11.5)	
Abortion						
No	326 (59.3)		310 (59.2)		16 (61.5)	
Yes	224 (40.7)		214 (40.8)		10 (38.5)	
Gestational diabetes						
No	491 (89.3)		475 (90.6)		16 (61.5)	
Yes	59 (10.7)		49 (9.4)		10 (38.5)	
Gestational hypertension						
No	536 (97.5)		512 (97.7)		24 (92.3)	
Yes	14 (2.5)		12 (2.3)		2 (7.7)	
Social support		29.5 (3.8)		29.7 (3.8)		26.7 (3.4)
Objective social support		12.4 (2.1)		12.5 (2.0)		11.3 (2.5)
Subjective social support		9.4 (1.8)		9.4 (1.8)		8.6 (1.2)
Availability of support		7.7 (1.8)		7.8 (1.9)		7.1 (1.4)
EPDS scores (T1)		7.6 (3.7)		7.6 (3.8)		7.0 (2.1)
EPDS scores (T2)		6.4 (3.5)		6.2 (3.3)		10.5 (3.2)
EPDS scores (T3)		6.2 (4.5)		5.4 (3.3)		18.4 (4.5)

*SD* Standard deviation, *T1* Late pregnancy (32 ± 4 weeks gestation), *T2* 1 week postpartum, *T3* 6 weeks postpartum, *EPDS* Edinburgh postnatal depression scale

### Identification of trajectories

The fit indices of the models generated through LGMM are reported in Table 2. The four- class solution was excluded given that the solution had one class comprising less than 10 women [27]. The AIC and BIC values suggested that the three-class solution yielded the best fit while the entropy value favoured the two-class solution. Compared with the two-class solution, the three-class solution produced one new subgroup (31.1%) characterized by the lowest and consistently declining depressive symptoms. However, the entropy was lower (0.563), indicating a poor latent classification quality. Taking these into consideration, the two-class solution was chosen as the optimal solution. The indices in the diagonal in Table 3 show that the classification accuracy was acceptable, with positive predictive values ranging from 89.7 to 97.1%.

**Table 2:** Fit indices of the for the LGMM models of EPDS, for increasing number of classes (1 to 4)

*No. of classes*		*AIC*	*BIC*	*BLRT*	*VLMR*	*Entropy*	*N per class*
1		7893.001	7905.931	–	–	–	–
**2**		**7780.155**	**7810.325**	***p*** **< .001**	***p*** **< .001**	**.862**	**524/26**
3		7726.320	7773.729	*p* < .001	*p <* .001	.563	360/19/171
4		7696.551	7761.200	*p <* .05	*p <* .05	.641	19/357/169/5

Bold indicates the selected category

*Abbreviations*: The values reported in this table are hypothetically derived for illustrative purposes. *AIC* Akaike information criterion, *BIC* Bayesian information criterion, *BLRT* Bootstrap likelihood ratio test, *VLMR* Vuong-Lo-Mendell-Rubin test

**Table 3:** Most likely latent class membership (row) by latent class (column)

Model	Decreasing	Increasing
Decreasing	0.971	0.029
Increasing	0.103	0.897

**Fig. 2:**
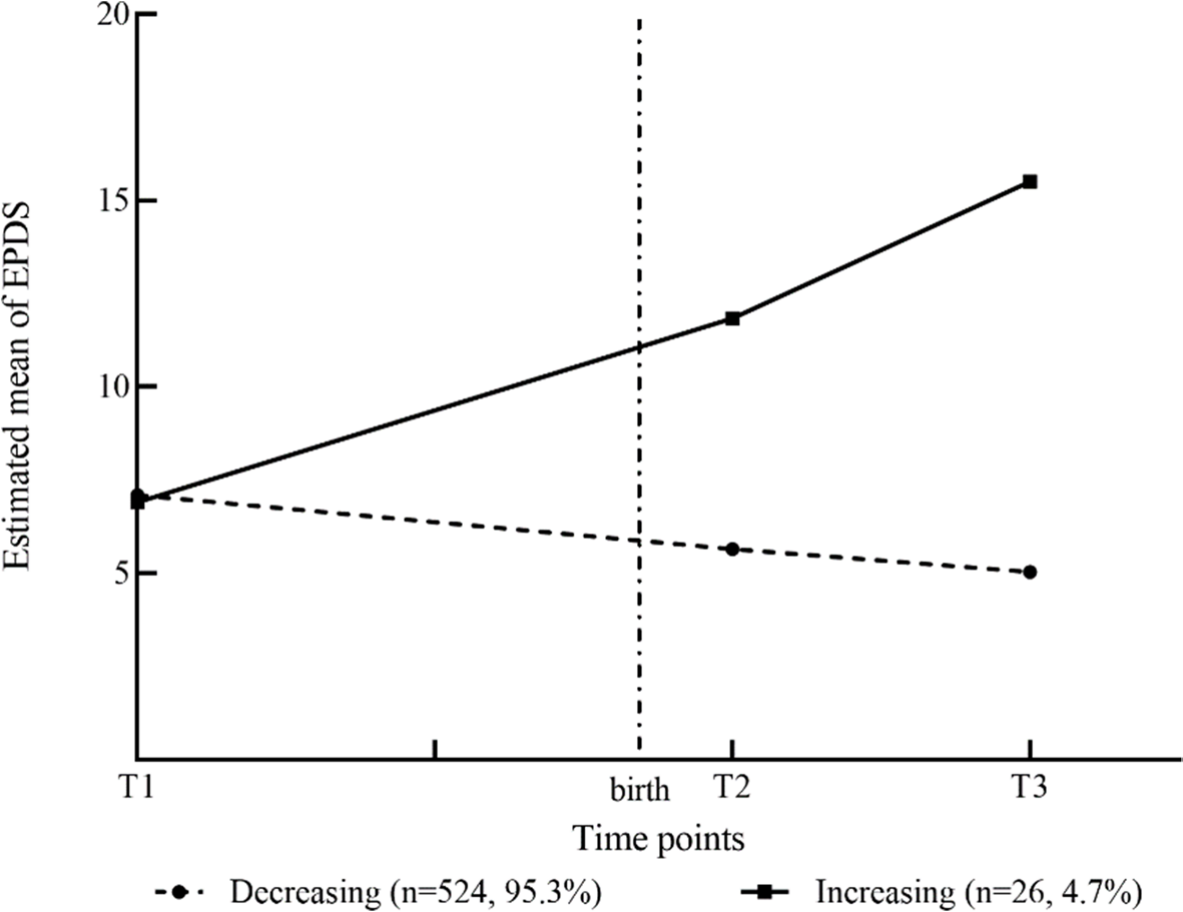
Estimated means for 2-class solution of EPDS (*n* = 550). T1: 32 ± 4 weeks gestation, T2: 1 week postpartum, T3: 6 weeks postpartum

The (small) increasing class is very homogeneous (close to zero random intercept), as shown in Additional file 3. Thus, all having the same expected trend, while in the other class individuals vary more around their mean trend. The estimated mean EPDS scores of women allocated to the two trajectories are presented in Fig. 2. The decreasing trajectory, with a estimated mean baseline score of 7.09, decreased to 5.64 at one-week postpartum and stabilized at 5.03 at six-weeks postpartum. The increasing group was characterised by no depressive symptoms at recruitment (estimated mean = 6.90), but increasing depression scores at one- and six-weeks postpartum (estimated mean = 15.50). The minority of the sample was allocated to class 2 (*n* = 26, 4.7%), with 95.3% of women (*n* = 524) allocated to the decreasing group.

### Predictors of trajectories

The results of the logistic regressions (reported in Table 4) indicated that the level of objective social support was important, greater overall levels of objective social support reduced the chances of worsening depression (OR = 0.76, 95%CI: 0.62–0.95). Greater overall levels of subjective social support at baseline decreased the odds of being assigned to the “increasing” group (OR = 0.81, 95%CI: 0.67–0.98). Participants with a history of smoking and alcohol use prenatally were 6.7 (95%CI: 1.73–26.02) and 3.35 (95%CI: 1.08–10.40) times more likely to belong to the “increasing” group. Mothers who reported prenatal gestational hypertension were 5.33 times more likely (95%CI: 1.07–24.49) to belong to the “increasing” group.

**Table 4:** Predictors of the latent trajectory group for perinatal depression symptom based on the logistic regression (reference class: decreasing)

Variables	Increasing (*n* = 26, 4.7%)		
	*OR*	95%*CI*	*p*
Age	1.05	0.95–1.15	0.352
Location			
Rural	1.01	0.44–2.32	0.974
City	ref		
Educational level			
≤ 9 years	1.05	0.35–3.19	0.928
10–12 years	0.46	0.13–0.60	0.222
13–16 years	ref		
Monthly income			
< ¥5000	1.69	0.72–3.40	0.230
≥ ¥5000	ref		
History of smoking			
Yes	6.70	1.73–26.02	0.006
No	ref		
History of alcohol			
Yes	3.35	1.08–10.40	0.037
No	ref		
Exercise during pregnancy			
Yes	0.86	0.25–2.94	0.810
No	ref		
Abortion			
Yes	1.12	0.49–2.48	0.810
No	ref		
Gestational diabetes			
Yes	1.60	0.53–4.83	0.402
No	ref		
Gestational hypertension			
Yes	5.33	1.07–24.49	0.041
No	ref		
Social support	0.84	0.77–0.93	0.001
Objective social support	0.76	0.62–0.95	0.015
Subjective social support	0.81	0.67–0.98	0.028
Availability of support	0.83	0.62–1.10	0.183

A simple logistic regression was performed, using only one predictor variable at a time

*OR* Odds ratio, *CI* Confidence interval

## Discussion

### Two heterogeneous subgroups predict the development of perinatal depression

LGMM was used to examine the heterogeneity of perinatal depressive symptoms from an individual-centred perspective and subsequently, the subtype characteristics of different prenatal and postnatal trajectories were described. In addition, we specified a Poisson distribution for the outcome variable. When a Poisson distribution was not specified, (thus, assuming normality), different classes emerged and with worse fit indices value, resulting in invalid findings, model misinterpretations, and possible non-intended practical implications [45].

The findings indicated that perinatal depression is heterogeneous and can be categorised based on specific characteristics of the individual. Specifically, the course of perinatal depression can be classified into two heterogeneous sub-groups or cohorts: “decreasing” and “increasing”; that is, two trajectories of PND were identified in this study. The majority of women (95.3%) were in a very stable state. They maintained a good mood throughout the perinatal period (EPDS scores were stable at a low level). This means that the majority of women remain at low risk of developing depressive symptoms during the perinatal period. This finding is consistent with previous research results [26–28]. However, the baseline EPDS scores in this study are higher than those observed in more developed countries. This may be related to different economic levels and living conditions between countries. Nonetheless, the severity of PND symptoms may significantly change over time in any population. In this study, 4.7% of women reported elevated PND symptoms over time, which is in contrast to previous studies [27, 46–48]. For example, a study of 425 pregnant women in South Africa found two depression trajectories [48]. The reason for this difference could be that the study in South Africa focused on low-income women, and a lack of food and living resources might affect the trajectory of perinatal depression. Moreover, some studies have reported the existence of a chronic and persistent high-level depression trajectory [27]. In this study, only 37% of the subjects lived with their partners and nearly 40% of the subjects were unmarried. In contrast, in the current study, more than 95% of women were married, and thus, would have more support from their partners, which has a positive effect on depressive symptoms [47]. In contrast, a long-term study conducted in Norway found no increasing trajectory, only a decreasing and lower EPDS trajectory [46]. This may be because women in that study had a higher education level, which may reduce the risk of depressive symptoms.

### Demographic characteristics of the two perinatal depression classes

The findings also suggest that that women with a history of smoking, alcohol use and gestational hypertension were more likely to experience worsening depressive symptoms. Passive smoking during pregnancy can lead to adverse pregnancy outcomes, including preterm birth, low birth weight, congenital disabilities, sudden infant death syndrome, and neurological and respiratory diseases [49, 50]. Data from the WHO data indicate that the rate of active smoking among Chinese women is low compared to European and American women. The rate of smoking among women aged over 15 years (after age standardization) is 20.7% in Europe, 12.4% in the Americas, and 1.9% in China [51]. Smoking during pregnancy is much less common in low- and middle-income countries, with an overall prevalence of 1.3% (range 0–15%), as estimated from the 2001–2012 Demographic and Health Survey data from 54 countries [52]. In contrast, Chinese women are fundamentally victims of passive smoking [51]. There is evidence that prenatal alcohol exposure at moderate and higher levels increase the odds of child neurobehavioural problems [53], including behaviour, aggression, attention, social functioning [54] and emotional problems [55]. In addition, the adverse effects of high prenatal alcohol exposure are more likely to occur in the offspring of women with a lower socio economic status (SES) than of those with a higher SES [56, 57]. Overall, the history of high levels of prenatal smoking and alcohol exposure can lead to adverse pregnancy outcomes, thereby increasing women’s levels of anxiety and depression. Therefore, pregnant women or women preparing for pregnancy should avoid smoking and drinking behaviors to reduce the effects of alcohol on physical, mental and fetal health.

Equally, pregnancy-induced hypertension has a considerable impact on both the mother and baby, leading to various obstetric and perinatal complications. Approximately 70% of pregnant women with hypertension during pregnancy develop preeclampsia, which has a worldwide incidence of 2 to 8%, or 2 to 16% in developing countries [58]. It is responsible for approximately 63,000 maternal deaths each year and is the principal cause of maternal and newborn deaths [59]. Pregnancy-induced hypertension increases anxiety about the outcome of a pregnancy. Thus, the negative effects of smoking history and pregnancy-induced hypertension in this study are easily understood. Such negative stimulation significantly predicts depression, and the more negative sexual events, the higher the likelihood of depression [60–63].

### Predictive effects of social support on perinatal depression

As an essential component of external resources, social support has consistently been positively correlated with psychological health [64, 65]. According to the stress-buffering model, social support may mitigate the psychological impact of a stressful event on mental health by attenuating the stress appraisal response [66]. Women tend to rate the harmful effects of depression and anxiety lower if they perceive more social support, thus reducing the impact of stressful events and leading to less negative emotions [67]. In the current study, the lower the level of subjective and objective social support, the more likely it is that depression will worsen. To date, there have been few studies examining the outcomes of subjective social support. For example, anecdotal evidence suggests that persistent major depression can weaken a patient’s subjective social support. When depressive symptoms are alleviated, social support levels will be significantly increased [68], an observation that is consistent with the current results where subjective social support was found to be a protective factor for perinatal depression. Furthermore, we propose that the more subjective support that is available, the more likely it is that a pregnant woman will be emotionally stable both before and after giving birth. Chinese research on subjective social support also shows that individuals with higher levels of subjective social support are more confident, feel more respected, understood and supported, and have fewer emotional disorders [69, 70]. Recent studies have also shown that a lack of objective support (such as material support, network support, marriage, and family support) for postpartum patients with depressive symptoms is detrimental [71]. This suggests that caring, support and encouragement from family and friends during pregnancy is the best way to relieve low mood and avoid the development of depressive symptoms and episodes.

### Intervention implications for perinatal depression

The U.S. Preventive Services Task Force recommends that women be screened for depression during pregnancy and postpartum care [72]. The current results suggest that women with low social support levels, a history of smoking, alcohol use or gestational hypertension are at high risk perinatally. It is essential to provide timely perinatal mental health education for pregnant women, particularly those at high risk. This education process would enable women to understand the occurrence, development and prevention of perinatal depression. Pregnant women, particularly those at high risk of perinatal depression, would be empowered to monitor their emotions, screen for mood swings and depressive symptoms, and seek psychological interventions from professional, medical and health institutions in a timely fashion.

### Limitation

This study’s limitations are as follows: 1) self-report questionnaire may have an impact on depression; 2) only the high-risk period was investigated: from the third trimester to 6 weeks postpartum: future studies should study the mood of perinatal depression (from pregnancy to 1 year postpartum) entirely, to characterise the trends of perinatal depression over the entire pregnancy; 3) the demographic and related characteristics data collected in this study are not comprehensive, and other factors may exist which augment the overall outcomes Future studies should include more variables such as women’s personality, and interpersonal relationships can be investigated to explore their specific impact on the development of depression; 4) the covariates only distinguished between belonging to either of the classes, but not how covariates may have impact on the (heterogeneous) EPDS levels within the largest class.

## Conclusion

This study has identified two trajectories of perinatal depression and the factors associated with each trajectory. The results demonstrated that higher social support is a protective factor for perinatal depression, while smoking, alcohol use history and prenatal hypertension during pregnancy are risk factors. As an extension of this research, it may be possible to achieve early identification of women at high risk of perinatal depression. In order to improve the depression symptoms of pregnant women, more attention should be paid to individuals with risk factors. Women with emotional and family/relationship problems should receive more social support. In future studies, these factors can be incorporated into a screening tool to identify women at risk of perinatal depression.

## Supplementary Information

**Additional file 1.** EPDS distribution histogram (T1: *n* = 550, T1: *n* = 487, T3: *n* = 447).

**Additional file 2.** Mplus Syntax.

**Additional file 3.** Parameter estimates of the final two class.

**Additional file 4.** Demographic information and questionnaires.

## Data Availability

The data used and analyzed during the study are available from the corresponding author if the request is reasonable.

## Abbreviations

PND: Perinatal depression
WHO: World Health Organization
AIC: Akaike information criteria
BIC: Bayesian information
BLRT: Bootstrap likelihood ratio test
VLMR: Vuong-Lo-Mendell-Rubin test
EPDS: Edinburgh Postnatal Depression Scale
SSRS: Social Support Rating Scale
LGMM: Latent variable mixed growth model
OR: Odds ratio
CI: Confidence interval
