# Supplementary material for: Distinct trajectories of perinatal depression in Chinese women: application of latent growth mixture modelling
Source: BMC Pregnancy Childbirth. 2022 Jan 10;22:24. doi: 10.1186/s12884-021-04316-0 (PMC8751241; doi:10.1186/s12884-021-04316-0)

**Additional file 1**: EPDS distribution histogram (T1: n=550, T1: n=487, T3: n=447).

In addition, the results of the Kolmogorov-Smirnov One-Sample Test proved that *p* < 0.05 for the three times data, indicating that the data were not normally distributed.


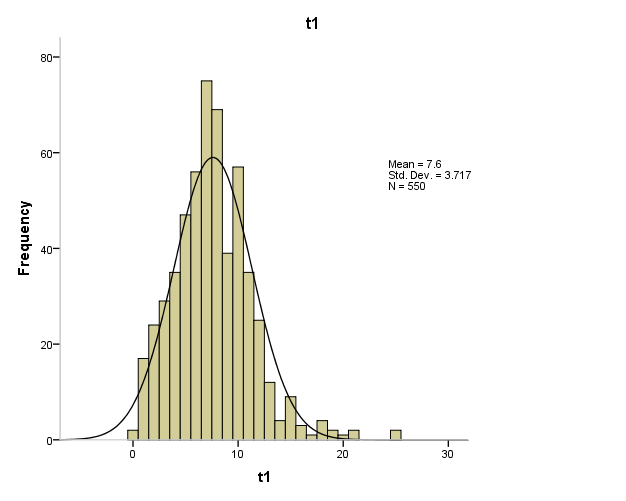


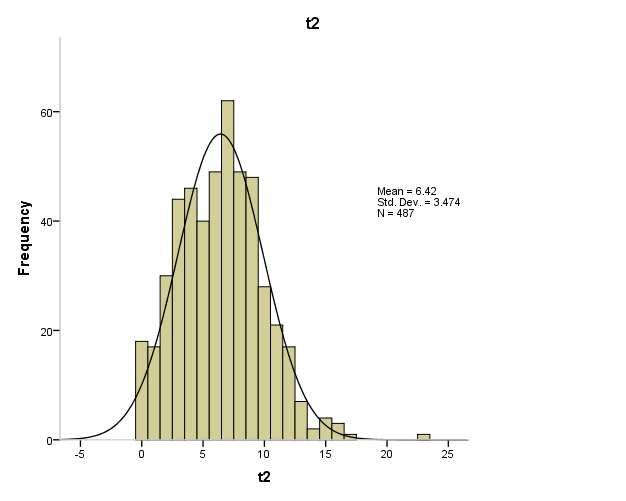


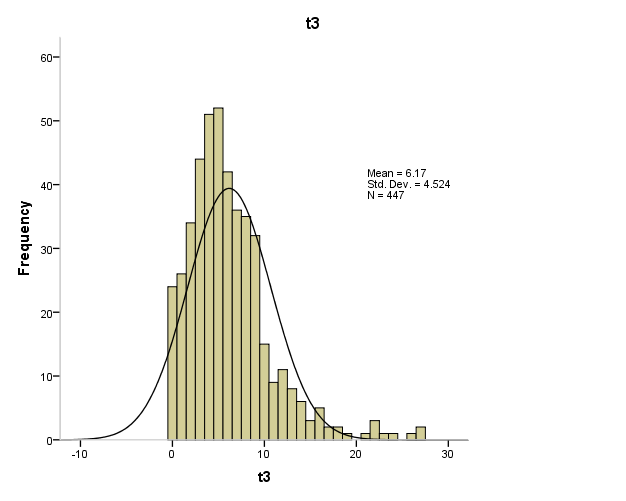

Supplement: Supplementary file 1 — Additional file 1. EPDS distribution histogram (T1: n = 550, T1: n = 487, T3: n = 447). [file 12884_2021_4316_MOESM1_ESM.docx]
