# Supplementary material for: Distinct trajectories of perinatal depression in Chinese women: application of latent growth mixture modelling
Source: BMC Pregnancy Childbirth. 2022 Jan 10;22:24. doi: 10.1186/s12884-021-04316-0 (PMC8751241; doi:10.1186/s12884-021-04316-0)
Supplement: Supplementary file 2 — Additional file 2. Mplus Syntax. [file 12884_2021_4316_MOESM2_ESM.docx]

**Additional file 2:** Mplus Syntax:

DATA:

FILE IS C:\Users\lan'lan\Desktop\EPDS.dat;

VARIABLE:

MISSING ARE ALL (-99);

NAMES ARE t1 t2 t3;

USEVAR=t1 t2 t3;

COUNT = t1 t2 t3;

Classes=c(2);

ANALYSIS:

Type=mixture;

Starts=200 10;

Processor=2;

ALGORITHM=INTEGRATION;

MODEL:

%overall%

i s | t1@0 t2@1 t3@1.5;

s@0;

%C#1%

i;

%C#2%

i;

Output:tech11 tech14;

plot:type is plot3;

series=t1 t2 t3( * );

savedata:save=cprob;

file is 2.csv;
