# Supplementary material for: Distinct trajectories of perinatal depression in Chinese women: application of latent growth mixture modelling
Source: BMC Pregnancy Childbirth. 2022 Jan 10;22:24. doi: 10.1186/s12884-021-04316-0 (PMC8751241; doi:10.1186/s12884-021-04316-0)
Supplement: Supplementary file 3 — Additional file 3. Parameter estimates of the final two class. [file 12884_2021_4316_MOESM3_ESM.docx]

**Additional file 3**. Parameter estimates of the final two class.

|  |  | Estimate | S.E. | t | *p* |
| --- | --- | --- | --- | --- | --- |
| Latent class 1 (n=524) |  |  |  |  |  |
| Means | I | 1.959 | 0.025 | 79.497 | 0.000 |
|  | S | -0.229 | 0.023 | -9.935 | 0.000 |
| Variances | I | 0.154 | 0.017 | 9.213 | 0.000 |
|  | S | 0.000 | 0.000 | 999.000 | 999.000 |
| Latent class 2 (n=26) |  |  |  |  |  |
| Means | I | 1.931 | 0.079 | 24.524 | 0.000 |
|  | S | 0.540 | 0.127 | 4.262 | 0.000 |
| Variances | I | 0.018 | 0.012 | 1.445 | 0.148 |
|  | S | 0.000 | 0.000 | 999.000 | 999.000 |

*Note*: variance of the slope was fixed to zero, I: intercept, S: slope.
