# Supplementary material for: Distinct trajectories of perinatal depression in Chinese women: application of latent growth mixture modelling
Source: BMC Pregnancy Childbirth. 2022 Jan 10;22:24. doi: 10.1186/s12884-021-04316-0 (PMC8751241; doi:10.1186/s12884-021-04316-0)
Supplement: Supplementary file 4 — Additional file 4. Demographic information and questionnaires. [file 12884_2021_4316_MOESM4_ESM.docx]

**Demographics**

**ID:**

**Age:**

**Gestational age：**

**Location: 🗖City 🗖Rural**

**Educational level： 🗖≤9 years 🗖10-12 years 🗖13-16 years**

**Monthly income： 🗖 <5000￥ 🗖≥5000￥**

**History of smoking： 🗖 No 🗖 Yes**

**History of alcohol: 🗖 No 🗖 Yes**

**Exercise** **during pregnancy: 🗖 No 🗖 Yes**

**Abortion: 🗖0 🗖 1 🗖 >1**

**Gestational diabetes: 🗖 No 🗖 Yes**

**Gestational hypertension: 🗖 No 🗖 Yes**

**Scale**

**1. Edinburgh postnatal depression scale, EPDS. ( See refs. [35])**

**2. Social Support Rating Scale, SSRS. ( See refs. [40])**
